# DECK: Distance and environment-dependent, coarse-grained, knowledge-based potentials for protein-protein docking

**DOI:** 10.1186/1471-2105-12-280

**Published:** 2011-07-11

**Authors:** Shiyong Liu, Ilya A Vakser

**Affiliations:** 1 Biomolecular Physics and Modeling Group, Department of Physics, Huazhong University of Science and Technology, Wuhan 430074, Hubei, China; 2 Center for Bioinformatics and Department of Molecular Biosciences, The University of Kansas, Lawrence, KS 66047, USA

## Abstract

**Background:**

Computational approaches to protein-protein docking typically include scoring aimed at improving the rank of the near-native structure relative to the false-positive matches. Knowledge-based potentials improve modeling of protein complexes by taking advantage of the rapidly increasing amount of experimentally derived information on protein-protein association. An essential element of knowledge-based potentials is defining the reference state for an optimal description of the residue-residue (or atom-atom) pairs in the non-interaction state.

**Results:**

The study presents a new Distance- and Environment-dependent, Coarse-grained, Knowledge-based (DECK) potential for scoring of protein-protein docking predictions. Training sets of protein-protein matches were generated based on bound and unbound forms of proteins taken from the DOCKGROUND resource. Each residue was represented by a pseudo-atom in the geometric center of the side chain. To capture the long-range and the multi-body interactions, residues in different secondary structure elements at protein-protein interfaces were considered as different residue types. Five reference states for the potentials were defined and tested. The optimal reference state was selected and the cutoff effect on the distance-dependent potentials investigated. The potentials were validated on the docking decoys sets, showing better performance than the existing potentials used in scoring of protein-protein docking results.

**Conclusions:**

A novel residue-based statistical potential for protein-protein docking was developed and validated on docking decoy sets. The results show that the scoring function DECK can successfully identify near-native protein-protein matches and thus is useful in protein docking. In addition to the practical application of the potentials, the study provides insights into the relative utility of the reference states, the scope of the distance dependence, and the coarse-graining of the potentials.

## Background

Protein-protein interactions are a key element of life processes. Thus better understanding of these interactions, coupled with our ability to model them, is essential for the fundamental knowledge of their biology and the multitude of biomedical applications.

Computational approaches to structural determination of protein-protein complexes (protein-protein docking) typically involve two steps: the global, often low-resolution, search within a computationally feasible timeframe to detect a set of matches that includes at least one near-native structure (scan stage), and the local refinement of the matches from the scan stage that may involve more computationally expensive protocols. Such refinement often includes scoring aimed at improving the rank of the near-native structure relative to the false-positive matches.

Knowledge-based potentials [1,2], physics-based potentials [3], and the hybrid potentials [4-6] have been shown to perform successfully in protein-protein docking benchmark tests. However, the limited ranking ability of the current scoring functions in CAPRI [7] suggests that much work still has to be done.

In structure prediction of individual proteins, the knowledge-based scoring functions gained significant popularity [8-10]. It has been shown that knowledge-based pairwise atomic potentials perform better than the physics-based potentials in the near-native structure refinement [11].

An essential element of knowledge-based potentials is defining the reference state for the optimal description of residue-residue (or atom-atom) pairs in the non-interaction state. For protein-protein interactions, generally, there are three methods of defining the non-interaction state. The first one is based on the large-distance cutoffs (e.g., DFIRE [12], DCOMPLEX with DFIRE-based potential [13], DOPE [14], and volume correction [15,16]), the second one is based on random mixing of residue or atom types (e.g., KBP [17], and DBD-Hunter [18]), and the third one is based on false-positive matches/decoys (e.g., RAPDF [19], PIPER [20], and DARS [2]). Our approach utilizes reference states based on protein-protein decoys. It was shown that the long-range cooperative interactions [21] play an important role in protein-protein association. However, they are difficult to model based on contact or physics-based potentials. On the other hand, the coarse-grained distance-dependent potentials are a simple way to capture the long-range residue-residue interaction. In this paper we present a new Distance- and Environment-dependent, Coarse-grained, Knowledge-based (DECK) potential for scoring of protein-protein docking predictions.

## Results

**Figure 1:**
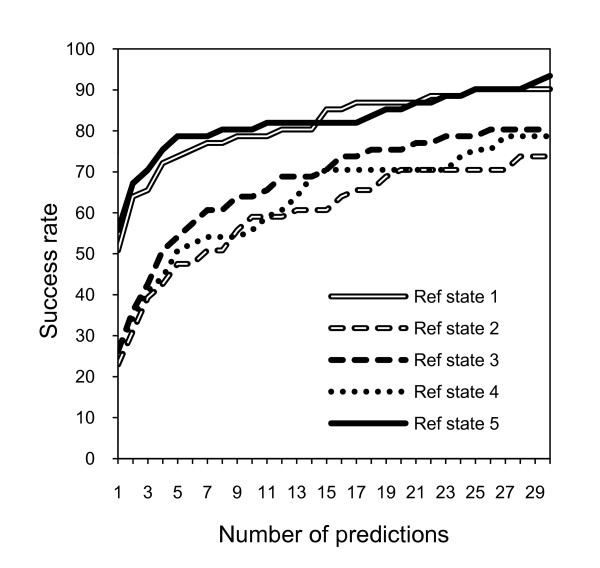
**Comparison of scoring results based on five reference states**. The success rates were determined on GRAMM-X docking decoys, as the percentage of complexes with at least one hit ranked in top N matches. A hit is defined as a match with ligand RMSD from the native structure <5 Å.

Coarse-grained statistical potentials were developed, based on pseudo-atoms at the geometric center of the side chains, with five different reference states. The potentials were trained on sets of unbound and bound protein-protein complexes (see Methods). To select the optimal reference state, the scoring functions were tested on GRAMM-X decoy set [22]. The success rate for each scoring function for the 61 complexes in the set is shown in Figure 1. The success rate was calculated as the percentage of complexes with at least one hit ranked in top N. A hit was defined as a match with ligand RMSD <5 Å. The success rates in Figure 1 provide a clear comparison of the five reference states, with the reference state 5 yielding the highest success rates overall, especially for the smaller top N values. Thus, further results in this study were obtained with the potentials based on this reference state.

**Figure 2:**
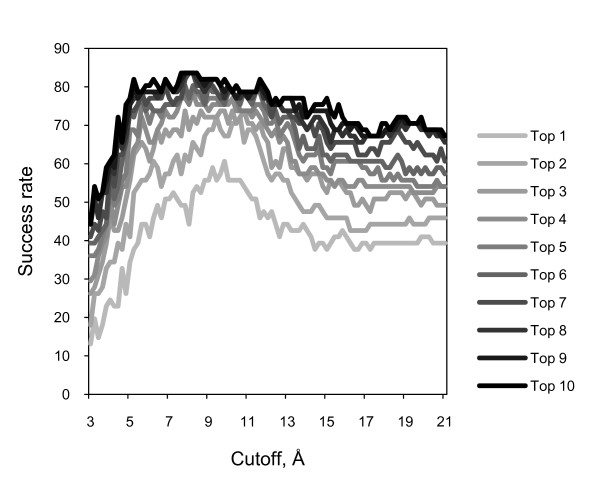
**Cutoff effect on the DECK potential**. The success rates of scoring, based on reference state 5 with different cutoff values, were obtained on GRAMM-X docking decoys. The cutoffs were tested with 0.2 Å step. The success rates were calculated as the percentage of complexes with hits (ligand RMSD <5 Å) in top N predictions, for different N values.

Our potentials are distance-dependent by design. In the development of distance-dependent potentials, the choice of the distance cutoff is an important consideration. Earlier studies investigated the cutoff effect in protein-protein energy landscapes [23]. For a long-range potential, such as soft Lennard-Jones, 14 Å cutoff was suggested. This value is close to the cutoff 15.5 Å in DFIRE [12]. In an iterative knowledge-based scoring function for protein-protein recognition, cutoff distance was set to 10 Å [24]. In the current study, for the scoring function with the reference state 5, cutoffs from 3.2 to 20.8 Å were used to check the cutoff effect on the success rate for the GRAMM-X decoys. The success rates were calculated for a set of top N criteria (Figure 2). The results show a decrease of the success rate for cutoffs >10 Å. This value is close to the cutoff values in ITScore [24]. The cutoff between 8 and 10 Å has little effect on the success rate. Thus, along with the distance-dependent potentials, we tested a contact potential, based on the reference state 5, which included a single 0 - 8Å bin.

**Figure 3:**
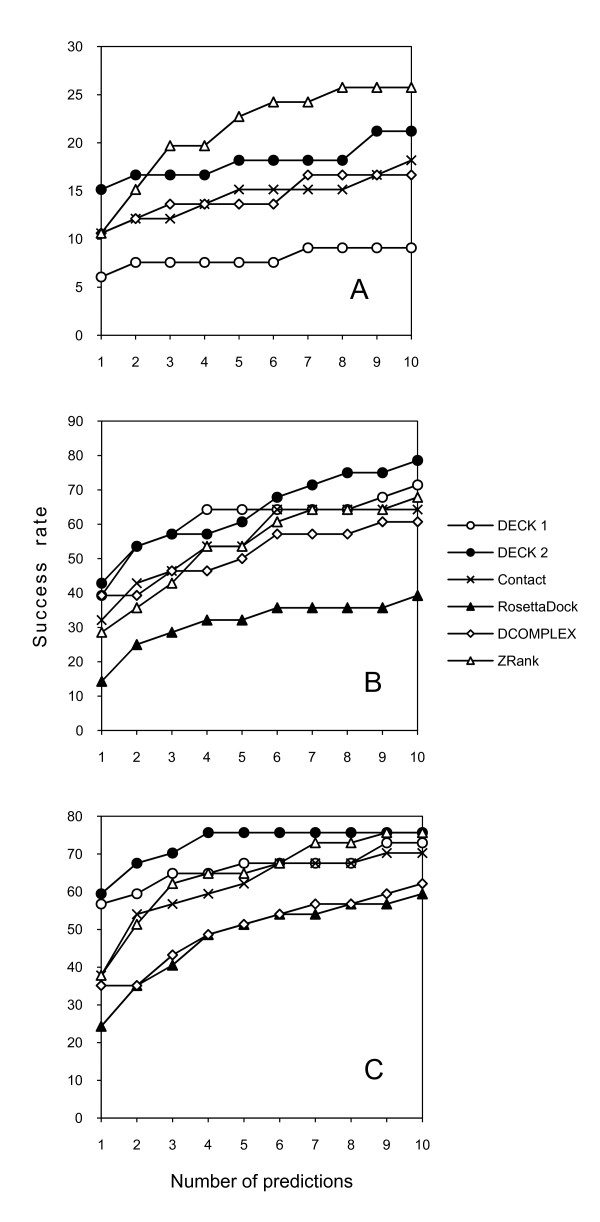
**Test on ZRANK and RosettaDock decoys**. DECK versions 1 and 2 are based on the reference state 5, and trained on set 1 and 2, correspondingly. The success rate was calculated as the percentage of complexes with at least one hit ranked in top N predictions. The definition of the hit is according to the test. (A) Test on ZRANK docking decoys. A hit is defined as a match with interface RMSD <2.5 Å. The ZRANK score and RMSD values were taken from the score file included with the decoys. (B) Test on RosettaDock decoys, with a hit defined as a match with ligand RMSD <5 Å, and (C) with a hit defined as a match with ligand RMSD <10 Å. The RosettaDock scores and RMSD values were taken from the score file included with RosettaDock decoys. DCOMPLEX and ZRANK scores were calculated locally.

The potentials were tested on the ZDOCK3.0+ZRANK Decoys developed in Weng's lab [25]. ZDOCK3.0 [1] implements FFT docking based on shape complementarily, electrostatics, and pairwise contact potentials. ZRANK [5] is an optimized energy function, which includes van der Waals, electrostatics and pairwise atomic contact energy. The dataset included 84 complexes with 54,000 decoys each. At least one near-native hit (a match with the interface C^α ^RMSD <2.5 Å) was present in 66 complexes. The tested potentials were: DECK 1 and DECK 2 (reference state 5, training sets 1 and 2, correspondingly), Contact Potential (trained on set 2), and DCOMPLEX. The results were compared with ZRANK values from the score file in the decoys set. The success rates are shown in Figure 3A. Overall, ZRANK showed the best results, except DECK 2 in the top 1 predictions. DECK 2 was better than Contact Potential and DCOMPLEX for all top N predictions.

A test was also performed on RosettaDock [4] unbound docking decoy set from Gray lab. The set includes 54 complexes. Each complex has top 200 structures from the global search based on unbound structures with rebuilt side chains. This decoy set represents another important facet of protein docking. The ZDOCK3.0+ZRANK set has the rigid body docking output, which typically contains a large number of matches for further structural refinement. The RosettaDock set contains the structures with optimized side-chain conformations, representing an expected output of a flexible structure refinement. Such a refinement is computationally expensive and thus has a significantly smaller number of matches, which are meant to be structurally more accurate than the rigid-body docking output.

DECK 1 and 2, and Contact Potential were tested and compared with RosettaDock, DCOMPLEX and ZRANK score values. The RosettaDock score values were obtained from the file in the decoy set. The scores of DCOMPLEX and ZRANK were computed locally. With a hit defined as a match with ligand RMSD <5 Å, 28 of 54 complexes had at least one hit. The results are shown in Figure 3B. If the hit was redefined as a match with ligand RMSD <10 Å, 37 of 54 complexes in the decoy set had at least one hit. Figure 3C shows the results according to this definition. As the results indicate, in both cases, DECK 2 outperformed other potentials across all top N predictions.

**Figure 4:**
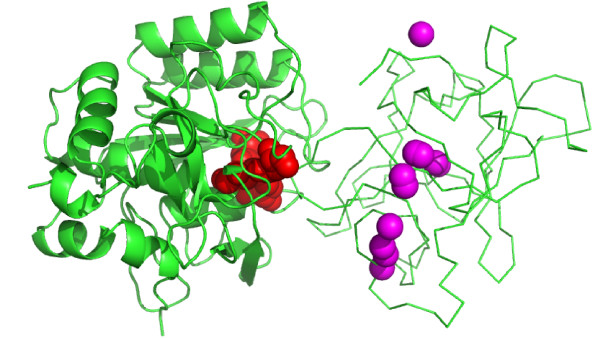
**Example of DECK scoring of protein-protein docking matches**. Top 10 models according DECK scores are shown for CAPRI target 32. The structures are shown in the correct (co-crystallized) position. Binding site residues on the receptor are in red. Magenta spheres are the geometric centers of the ligand in the top 10 predictions containing two acceptable models (see text for details).

An important activity in the field of protein-protein docking is a community-wide experiment on Critical Assessment of Predicted Interactions (CAPRI; http://www.ebi.ac.uk/msd-srv/capri). This experiment allows a comparison of different computational methods on a set of prediction targets (co-crystallized protein complexes with the structure of the complex unknown to the predictors). The community of predictors is provided with the coordinates of the separate components of the complex, which they use for the docking and scoring. After the models are submitted by the docking predictors, they are made available to 'scorer' groups to re-rank them and submit their own 10 best-ranking matches [7]. The DECK potential was tested in the CAPRI scoring experiment. According to the CAPRI assessment criteria, it identified two 'acceptable' models for target 32, four 'medium' models for target 40, four 'medium' and three 'acceptable' models for target 41, and one 'acceptable' model for target 46. Target 32 was a complex between subtilisin Savinase and α-amylase subtilisin inhibitor. The distribution of the top 10 models for this target is shown in Figure 4 (the best results for the target among twenty scoring teams).

The scoring procedure implementing DECK is available from the authors upon request (liushiyong@gmail.com).

## Discussion

The knowledge-based potentials improve modeling of protein complexes by taking advantage of the rapidly increasing amount of experimentally derived information on protein-protein association. The distance dependence of these potentials is supposed to provide a more accurate description of protein-protein interactions by taking into account the structural and physicochemical aspects of the interacting proteins within a broader scope than the immediate contact across the interface. The coarse-graining of the potentials makes them less sensitive to the structural inaccuracies of the proteins, which are unavoidable for unbound X-ray and potentially modeled proteins, especially in high-throughput applications to large interaction networks.

Five reference states for the coarse-grained, distance-dependent, knowledge-based potentials were used in this study. Similar reference states in earlier studies focused on protein structure prediction and protein folding [19,26,27]. We applied a similar form of the potential to protein-protein docking, redefining the reference states based on the non-native matches (docking decoys). The larger number of non-native matches models random protein-protein binding with reasonable accuracy. The long range interactions were accounted for by incorporating the structural environment of the interacting residues. Docking decoys were used as a reference state earlier in DARS potentials [2]. However, our method differs in three key points. The first one is the detailed form of the potential. DARS is based on the mole fraction potential, uniform reference state, and atomic contact potentials [28] (the random crystal reference state: the atom pairs are randomly exchanged). In our method, the reference states 1 and 2 also include the mole fraction terms. However, they also incorporate the probability of finding residue types at a certain distance [19]. The second point is the way to calculate the observed and the expected probabilities of residue pairs. The observed probability of DARS is based on the native structure. In our study, the observed probability based on the native structure made the results worse when tested on GRAMM-X decoys (data not shown). The main reason was the limited number of nonredundant protein-protein interfaces. So, in our approach the near-native matches were used instead of the native complexes. The DARS approach used 20,000 best scoring matches (shape complementarily only) for calculating the reference probabilities. We used ~160,000 best scoring matches without the near-native hits for calculating the expected probability in each case. The third point is the resolution. Our method is coarse-grained. Because in this work we do not integrate our potential in the FFT search, a direct comparison of the results is difficult. However, both studies show that the reference states based on decoys perform better than the ones based on mole fraction terms. Overall, the results show that the scoring function DECK can successfully identify near-native protein-protein matches and thus is useful in protein docking.

## Conclusions

Scoring of predicted protein-protein matches is important for identification of near-native structures in a pool of models. Knowledge-based scoring schemes improve modeling of protein complexes by taking advantage of the rapidly increasing amount of experimentally derived information on protein-protein association. A choice of the reference state for the description of non-interacting residue or atom pairs is an essential element of the knowledge-based potentials. The study presents a new potential for scoring of protein-protein docking predictions. Training sets of protein-protein matches were generated based on the bound and unbound proteins from the DOCKGROUND resource. Each residue was represented by a pseudo-atom in the geometric center of the side chain. To capture the long-range and the multi-body interactions, residues in different secondary structure elements at protein-protein interfaces were considered as different residue types. Five reference states for the potentials were defined and tested. The optimal reference state was selected and the cutoff effect on the distance-dependent potentials investigated. The potentials were validated on the docking decoys sets, showing better performance than the existing potentials used in scoring of protein-protein docking results. The study also provides insights into the relative utility of the reference states, the scope of the distance dependence and the coarse-graining of the potentials.

## Methods

### Training sets

The bound and the unbound complexes for the training sets were taken from the DOCKGROUND resource [22,29,30] (http://dockground.bioinformatics.ku.edu). The bound complexes were from the representative bound set and the bound part of the docking benchmark. The unbound complexes were from the docking benchmark. For all the complexes, the docking decoys were generated by GRAMM-X [31] scan (with no scoring and refinement). A match with RMSD of the ligand backbone atoms <5 Å was defined as the near-native one, comparable with CAPRI evaluation criteria [7]. With 160,000 matches per complex, 358 bound complexes from the representative set, and 71 bound complexes and 50 unbound complexes from the docking benchmark set had at least one near-native prediction. Two training sets were compiled: Training Set 1 (408 complexes) including 358 bound complexes from the representative set and 50 unbound complexes from the docking benchmark, and Training Set 2 (429 complexes) including 358 bound complexes from the representative set and 71 bound complexes from the docking benchmark. It is well known that existing protein-protein docking procedures perform differently on bound and unbound structures. Thus, it is interesting to see the difference between the knowledge-based potentials derived from the bound and from the unbound docking, especially with the potentials tested on the unbound docking decoys.

### Knowledge-based energy functions

It can be assumed that the probability of structural features at protein-protein interfaces follows the Boltzmann distribution [12,17,19,26,27,32-37]. For a residue-residue pair (*i*, *j*) at distance *d *across the interface, the contribution of binding energy *e*(*i*, *j*, *d*) can be estimated as:(1)

where *π*(*i*,*j*,*d*)_*obs *_and *π*(*i*,*j*,*d*)_*exp *_are the observed and the expected probability of the residue pair (*i, j*) at distance *d *respectively, and RT is set to 1.

The interaction distance was divided into 21 bins. Comparison with the contact potential (Figure 3) suggests that the larger number of bins enhances the performance of the potential. At the same time, increasing the number of bins beyond 21 would contradict the coarse-grained, residue-based nature of the potential.

Five reference states from the existing methodologies were defined. Each residue was represented by a pseudo-atom in the geometric center of the side chain (for GLY, the geometric center of the main chain). The distance between residues *i *and *j *was defined as the distance between their pseudo atoms. Atomic environment potential [38] was used to model multi-body interaction from pairwise contact potentials. To capture the long-range and the multi-body interactions, residues in different secondary structure environments [39,40] (helix, strand, and coil) at protein-protein interfaces was considered as different residue types. The total number of such types was 60 (20 amino acids in three secondary structure states). The secondary structure state was calculated by DSSP [41]. The eight DSSP secondary structure states are usually placed in three groups: helix (G, H and I), strand (E and B) and loop (all others). In our study, besides H and E, other states were designated as O. So the three secondary structure states were: H, E and O.

All residue-residue pairs were from protein-protein interfaces of the near-native matches or non-near native decoys. A residue was assigned to the interface if its centroid was within 30 Å of any residue centroid of the other docking partner. Different methods of calculating the probabilities in observed and expected states lead to different potentials. In the following part, we will discuss five different methods used to define the reference state.

### Reference state 1

The observed probability of residue pair (*i, j*) was defined as(2)

where *d *is the distance between residues *i *and *j*;(3)(4)

where *n*_*p *_is the total number of complexes; *n*_*m *_is the total number of near-native matches in each complex; the number of residue types (NRT) is 60; *g*_*p*__,__*m*_(*i*,*j*,*d*) is the total number of *i*, *j *pairs at distance *d *in near-native structure *m *of complex *p*.

The mole fraction of residue type *i *is defined as:(5)

where *N (i) *is the total number of type *i *residues at the near-native interface. The expected probability of residue pair (*i, j*) is defined as:(6)

The expected probability of residue pair (*i, j*) is estimated from the near-native matches. The expected probability of residue pair (*i, j*) was calculated in the same way as the observed probability using all decoys instead of the near-native matches in Eqs. (2-5).

### Reference state 2

This reference state is based on KBP potential [17]. The observed and the expected probabilities of residue pair (*i, j*) were calculated from near-native matches. The observed probability of residue pair (*i, j*) was defined as(7)

where *d *is the distance between residues *i *and *j*.

The expected probability of residue pair (*i*, *j*) is defined as:(8)

where *N*(*i*,*j*,*d*)_*obs*_, *N*(*d*)_*obs *_and mole fraction *χ*_*i *_are calculated according to Eqs. (3-5).

### Reference state 3

This reference state was proposed by Sippl [27]. The observed and the expected probabilities of residue pair (*i, j*) are calculated from near-native matches. The observed probability of residue pair (*i, j*) is defined as:(9)

where *σ *is set to 0.02.(10)

### Reference state 4

The observed and the expected probabilities of residue pair (*i, j*) were calculated from the near-native matches. The observed probability of residue pair (*i, j*) was defined as(11)

where *d *is the distance between residues *i *and *j*.

The expected probability of residue pair (*i, j*) at distance *d *was defined as:(12)

where *N*(*i*,*j*,*d*)_*obs *_and *N*(*d*)_*obs *_are calculated according to Eq. (3) and Eq. (4) respectively.

### Reference state 5

The observed probability *π*(*i*,*j*,*d*)_*obs *_and the expected probability *π*(*i*,*j*,*d*)_*exp *_of residue pair (*i, j*) were calculated from the near-native matches and the non-native decoys according to Eq. (11), respectively. The only difference between the observed probability *π*(*i*,*j*,*d*)_*obs *_and the expected probability *π*(*i*,*j*,*d*)_*exp *_of residue pair (*i, j*) are the objects of the statistics - the near-native matches for the former and the non-native decoys for the latter.

## Authors' contributions

IAV conceived the research and both authors designed it. SL carried out the calculations and both authors analyzed the results. The manuscript was drafted by SL and written/revised by both authors. Both authors have read and approved the final manuscript.

## Authors' information

SL is an associate professor at the Department of Physics at Huazhong University of Science and Technology, and IAV is the director of the Center for Bioinformatics and professor of Bioinformatics and Molecular Biosciences at The University of Kansas.
